# Enhanced Wound Healing With β-Chitosan-Zinc Oxide Nanoparticles: Insights From Zebrafish Models

**DOI:** 10.7759/cureus.69861

**Published:** 2024-09-21

**Authors:** Tharansia Ramachandran, Karthik Ganesh Mohanraj, Taniya Mary Martin, Meenakshi Sundaram K

**Affiliations:** 1 Department of Anatomy, Zebrafish Facility, Saveetha Dental College and Hospitals, Saveetha Institute of Medical and Technical Sciences, Saveetha University, Chennai, IND

**Keywords:** antimicrobial, nanotechnology, regenerative properties, wound healing, zebrafish, zinc oxide nanoparticles, β-chitosan

## Abstract

Introduction: Wound healing is a complex physiological process essential for the restoration of tissue integrity and function. Novel therapeutic approaches are urgently needed to enhance wound-healing outcomes. Nanotechnology, particularly zinc oxide nanoparticles, has shown promise due to its antimicrobial, anti-inflammatory, and regenerative properties. β-chitosan, derived from squid pens, possesses superior solubility and bioactivity compared to α-chitosan, making it a valuable biomaterial for biomedical applications. Through the integration of β-chitosan and zinc oxide nanoparticles, this study seeks to use the complementary properties of both substances to overcome present constraints in wound care treatments.

Methods: β-chitosan was extracted from squid pens and characterized for its molecular weight, degree of deacetylation, and solubility properties. Further characterization of the synthesized zinc oxide nanoparticles involved Fourier transform infrared spectroscopy to analyze chemical bonding and functional groups, ultraviolet-visible spectroscopy to determine optional properties such as band gap energy, X-ray diffraction spectroscopy to confirm the crystalline phase and calculate crystallite size, and the size was confirmed with the scanning electron microscope. Each technique provided complementary information, ensuring a comprehensive understanding of the synthesized nanoparticles' properties and their potential applications. Adult zebrafish (six to eight months old) were employed as a model organism due to their genetic similarity to humans and regenerative capabilities. Zebrafish were wounded and divided into treatment and control groups, with β-chitosan and β-chitosan-derived zinc nanoparticles treatments administrated at 50 µg/ml, while control groups received 0.05% phosphate buffer saline. The treatments, conducted in triplicate, enabled a comparative analysis of wound closure activity between β-chitosan-derived zinc nanoparticles' healing effects against standard and baseline treatments. Further, gene expression analysis on Bax, BCl-2, IL-2, IL-6, and tumor necrosis factor-alpha (TNF-a) was done using reverse transcriptase polymerase chain reaction.

Results: Characterization studies confirmed the successful synthesis of β-chitosan-derived zinc oxide nanoparticles and a crystalline structure corresponding to zinc oxide. Treatment with β-chitosan-derived zinc oxide nanoparticles significantly accelerated wound closure compared to controls and other treatment groups. Microscopic analysis demonstrated enhanced epithelialization, reduced inflammatory cell infiltration, increased collagen deposition, and improved tissue organization in wounds treated with β-chitosan-derived zinc oxide nanoparticles. Gene expression analysis revealed downregulation of inflammation-causing genes such as BCl-2, IL-2, IL-6, and TNF-a, hence it showed wound-healing activity. The results were statistically significant (p < 0.05).

Conclusion: β-chitosan-derived zinc oxide nanoparticles show promising potential as a novel therapeutic strategy for enhancing wound healing. The synergistic effects of β-chitosan and zinc oxide nanoparticles address multiple aspects of wound healing, including antimicrobial activity, inflammation modulation, and tissue regeneration. This study highlights the advantages of nanotechnology in wound care and underscores the need for further research to optimize nanoparticle formulations for clinical applications.

## Introduction

Wound healing is recognized as a critical physiological process, involving a complex series of events aimed at restoring the integrity of injured tissues [1]. These events encompass hemostasis inflammation, proliferation, and remodeling, all orchestrated to ensure efficient and complete healing. Despite the inherent capacity of the body to heal wounds, various factors such as infection, chronic diseases, and compromised immune function impede the healing process, leading to chronic wounds and significant morbidity [2]. Consequently, the development of novel therapeutic strategies to enhance wound healing has become paramount in clinical practice. In recent years, the field of nanotechnology has offered promising solutions for enhancing wound healing through the development of nanoparticles with unique properties. Among these, zinc oxide nanoparticles have garnered significant attention due to their remarkable antimicrobial, anti-inflammatory, and regenerative properties [3]. Zinc oxide, a multifunctional material, has been widely used in various biomedical applications owing to its biocompatibility and ability to interact with biological systems at the nanoscale. Conventional wound-healing treatments, including dressings, antibiotics, and surgical interventions, often face limitations such as insufficient antimicrobial activity, delayed healing, and the risk of antibiotic resistance [4]. These challenges underscored the need for innovative approaches that could address multiple aspects of the wound-healing process simultaneously. In this context, the integration of biopolymers such as chitosan with nanoparticles presented a promising avenue for enhancing wound-healing efficacy [5]. Chitosan, a natural polysaccharide derived from chitin, has been renowned for its biocompatibility, biodegradability, and bioactivity. It has been extensively studied for various biomedical applications, including wound healing. Chitosan's ability to form films, fibers, and hydrogels made it an attractive candidate for wound dressings and drug delivery systems. Furthermore, chitosan exhibited intrinsic antimicrobial properties, which could be further enhanced by chemical modifications. Among the different forms of chitosan, β-chitosan, extracted from squid pens, has gained attention due to its unique physicochemical properties. β-chitosan exhibits higher solubility and greater biological activity compared to its α-counterpart, making it a superior candidate for biomedical applications. Its unique molecular structure and higher degree of acetylation contribute to its enhanced bioactivity and potential in promising wound healing [6].

Zinc oxide nanoparticles are distinguished by their small size, large surface area, and distinctive physicochemical properties, which enable them to interact effectively with biological systems [7]. Zinc oxide nanoparticles demonstrated potent antimicrobial activity against a wide range of pathogens, including bacteria, fungi, and viruses. The antimicrobial property is primarily attributed to the generation of reactive oxygen species (ROS) and the release of zinc ions, which disrupt microbial cell membranes and interfere with their metabolic processes [8]. In addition to their antimicrobial effects, zinc oxide nanoparticles have shown anti-inflammatory cytokine production and reduced oxidative stress. These properties are crucial in the context of wound healing, where excessive inflammation could impede the healing process and lead to chronic wounds. Furthermore, zinc oxide nanoparticles have been reported to promote cellular proliferation and migration, essential components of the wound-healing process [9]. Given the promising properties of β-chitosan and zinc oxide nanoparticles, this study aimed to evaluate the effectiveness of β-chitosan-derived zinc oxide nanoparticles (β-Ch-ZnO-NPs) in promoting wound healing. The specific objectives included assessing the anti-inflammatory, antimicrobial, and regenerative effects of these nanoparticles [10]. To achieve these objectives, the zebrafish model was employed, an established organism for studying wound healing due to its remarkable regenerative capabilities and transparent embryos, which facilitated observation and analysis. The zebrafish (*Danio rerio*) has emerged as a valuable model organism in biomedical research, particularly for studying developmental processes, disease mechanisms, and regenerative medicine [11]. Moreover, zebrafish offer a high degree of genetic homology with humans, making them a relevant model for studying human diseases and therapeutic interventions [12]. One of the key advantages of using zebrafish in wound-healing studies is their rapid and robust regenerative capacity. Unlike mammals, zebrafish could efficiently regenerate various tissues, including skin, heart, and even parts of the central nervous system. This regenerative ability, combined with their transparent embryos, enabled detailed studies of wound-healing dynamics, cellular behaviors, and molecular mechanisms [13]. The use of zebrafish in research necessitated adherence to ethical guidelines to ensure their humane treatment and welfare. All experimental procedures involving zebrafish were conducted in accordance with institutional and national ethical standards. Proper housing, feeding, and care of the zebrafish were ensured to minimize stress and promote their well-being. Ethical considerations also included minimizing the number of animals used and employing alternative methods whenever possible [14]. The successful application of β-Ch-ZnO-NPs in promoting wound healing could have significant implications for clinical practice. By addressing key aspects of the wound-healing process, including antimicrobial activity, inflammation modulation, and tissue regeneration, these nanoparticles held promise for improving wound-healing outcomes [15]. Furthermore, the findings from this study could pave the way for the development of novel nanoparticle-based therapies for other tissue repair and regenerative medicine applications. The study aimed to investigate the potential of β-Ch-ZnO-NPs in enhancing wound healing using the zebrafish model. By leveraging the unique properties of β-chitosan and zinc oxide nanoparticles, and the advantages of the zebrafish model, the research sought to provide comprehensive insights into the mechanisms and efficacy of this novel therapeutic approach. The results of this study could contribute to the advancement of nanoparticle-based strategies for wound healing and broader biomedical applications [16]. Previous studies have demonstrated the potential of nanoparticles in biomedical applications. In the context of wound healing, nanoparticles offer several advantages. Their small size allows for better penetration into tissues, and their large surface area enables a higher load of therapeutic agents. Moreover, the surface properties of nanoparticles could be tailored to enhance their interaction with biological systems. For example, surface functionalization of nanoparticles with ligands or antibodies could target specific cells or tissues, thereby improving the efficacy of treatment [17].

The study aimed to evaluate the potential of β-Ch-ZnO-NPs in promoting wound healing using zebrafish. By leveraging the unique properties of β-Ch-ZnO-NPs, and the advantages of the zebrafish model, the research sought to provide comprehensive insights into the mechanisms and efficacy of this novel therapeutic approach. The successful application of β-Ch-ZnO-NPs in promoting wound healing provides significant implications for clinical practice, potentially leading to the development of novel nanoparticle-based therapies for wound healing and broader biomedical applications [18].

## Materials and methods

Synthesis of β-Ch-ZnO-NPs

β-chitosan was obtained from squids through a carefully controlled extraction procedure. Squid bones were cleaned, sun-dried for four days, ground, and sieved to an 80 mesh size. Fifty grams of this powder was determined by treating it with a 1 M hydrochloric acid solution (1:10 ratio) for three hours. The mixture was then filtered and washed until the chloride ions were removed. The residue was subsequently treated with 1 M sodium hydroxide (1:10 ratio), heated to 60°C, filtered, and washed until neutral. The resulting chitosan was dried at 70°C and stored at -80°C. To synthesize zinc nanoparticles using β-chitosan, a solution of zinc nitrate (0.1 mM) was prepared by dissolving it in deionized water. Similarly, a 0.5% solution of β-chitosan was also prepared. These two solutions were combined and mixed thoroughly under constant stirring. Next, a freshly prepared 0.1 M sodium borohydride solution was added dropwise to the mixture while stirring vigorously to reduce the zinc ions and produce β-chitosan-derived zinc nanoparticles. The stirring continued for 30 minutes to complete the reduction and stabilize the nanoparticles. The nanoparticle solution was then centrifuged at 10,000 rpm for 20 minutes to separate the nanoparticles from any unreacted materials and by-products. The supernatant was discarded, and the nanoparticles were washed several times with deionized water to remove any remaining reactants, ensuring the final product's purity and stability [19].

Characterization of β-Ch-ZnO-NPs

Following the synthesis of β-Ch-ZnO-NPs, characterization was done using analytical techniques, including scanning electron microscopy (JEOL JSM-IT 800, JEOL Ltd., Tokyo, Japan) and ultraviolet-visible (UV-vis) spectrophotometry (Shimadzu UV equipment, Shimadzu Corporation, Kyoto, Japan) employed to scan the nanoparticles, detecting any absorbance changes within the wavelength range of 200-700 nm. The particle size of β-chitosan-derived zinc nanoparticles was calculated using the Debye-Scherrer equation, where λ represents the X-ray wavelength, β is the full width at half maximum (FWHM), and θ is the Bragg’s angle. Fourier transform infrared spectroscopy (FTIR) (Agilent FTIR Spectrometers, Gurugram, India) using KBr pellets in the 500-4,000 cm⁻¹ range identified functional groups present in the β-chitosan extract responsible for reducing zinc ions to nanoparticles. X-ray diffraction (XRD) (Rigaku MiniFlex, Mumbai, India) analysis confirmed the crystalline structure and high purity of the β-Ch-ZnO-NPs, with sharper and narrower Bragg peaks observed in annealed samples, indicating enhanced crystallinity. These characterization techniques collectively provided comprehensive insights into the structural, morphological, and chemical properties of β-Ch-ZnO-NPs [20].

Zebrafish maintenance

The present study was approved by the Institutional Animal Ethical Committee, Saveetha Dental College and Hospitals, Saveetha Institute of Medical and Technical Sciences (SIMATS) (Approval No.: BRULAC/SDCH/SIMATS/IAEC/06-2023/15). Adult zebrafish (*Danio rerio*), AB strain (six to eight months old), procured from a local shop were maintained in a controlled environment, adhering to a 14-hour light/10-hour dark cycle to simulate natural conditions. They were fed a balanced diet twice daily to ensure proper nutrition and overall health. Water quality parameters, including pH, temperature, and dissolved oxygen levels, were regularly monitored and maintained within optimal ranges to provide a stable and healthy living environment for the zebrafish. The water was continuously filtered and aerated to maintain cleanliness and oxygenation. Regular partial water changes were performed to remove waste products and prevent the buildup of harmful substances. The zebrafish tanks were equipped with proper hiding places and enrichment items to reduce stress and promote natural behaviors. Additionally, the zebrafish were regularly inspected for signs of disease or distress, and any affected individuals were promptly isolated and treated to prevent the spread of illness within the populations [21].

Wound infliction

To inflict wounds on adult zebrafish, the fish were first anesthetized using an ice-cold water bath to reduce stress and discomfort. A standardized wound was then made on the dorsal fin with a sterile scalpel, ensuring uniformity across all subjects. The scalpel was sterilized before each use to avoid infection. Following the procedure, the zebrafish were placed in fresh, clean water to recover, with close monitoring for any signs of distress or complications. Recovery tanks were maintained with optimal water quality parameters, including pH, temperature, and dissolved oxygen, to facilitate healing. The fish were regularly observed to evaluate their behavior and overall health, with any adverse effects being promptly addressed. This meticulous approach to handling and post-wounding care ensured both the well-being of the zebrafish and the accuracy of the experimental results [22].

Treatment administration

Adult zebrafish were wounded and then placed in treatment and control groups (n = 6). Fish grouped with β-chitosan and β-Ch-ZnO-NPs were treated with 30 µg/ml of the corresponding compounds, while the control groups received 0.05% v/v phosphate buffer saline. The concentration was determined based on the toxicity dose (data not shown). Each treatment (controls and treatments) was performed in triplicates. A comparative investigation of the healing effects of β-Ch-ZnO-NPs against standard and baseline treatments was made possible by these different treatments, following the protocols previously reported [22].

Observation and imaging

Observation and imaging of the wound-healing process involved daily monitoring using a stereomicroscope to track the progress of wound closure and tissue regeneration. Randomly, fish from different groups were selected without bias and the images of the wound site were captured at specific time points, namely, three, seven, and 15 days post-wounding, to document and assess the healing progress. Wound closure (%) was calculated by the following formula: wound closure (%) = area of the wound on respective day/area of the wound on day 0. The data were represented as mean ± standard deviation, two-way ANOVA, and Bonferroni post-tests were used to analyze the statistical significance between the treatment groups with p < 0.05 [22].

Anti-inflammatory analysis

For the anti-inflammatory analysis, wound tissue samples were collected from the zebrafish at the end of the study (day 15) following the infliction of the wound. The expression levels of key inflammatory cytokines, especially Bax, BCl2, IL-2, IL-6, and tumor necrosis factor-alpha (TNF-a)* *were analyzed using quantitative polymerase chain reaction (PCR) [22]. The primers were designed and synthesized (Eurofins Genomics, Bengaluru, India), and total RNA was isolated (TRIzol method, Takara Bio, New Delhi, India). Respective complementary DNA (cDNA) was synthesized (Takara Bio) and quantitative PCR was analyzed (CFX 96 Touch Real-Time PCR detection system, Bio-Rad, Hercules, CA). This technique enabled the precise quantification of cytokine mRNA levels, providing insights into the inflammatory effects of β-Ch-ZnO-NPs and their potential role in promoting a conducive environment for wound healing. The data were represented as mean ± standard deviation and one-way ANOVA and Dunnett's multiple comparison tests were used to analyze the statistical significance between the treatment groups with p < 0.05.

Statistical analysis

As mentioned in the respective method section for separate assays, the data analysis was conducted using GraphPad Prism (GraphPad Software, San Diego, CA), a statistical software to ensure accurate and reliable results. A p-value of less than 0.05 was considered statistically significant [20-22].

## Results

In the results, statistical analysis revealed significant differences between the treatment and control groups in terms of wound-healing outcomes. Zebrafish treated with β-Ch-ZnO-NPs showed better wound closure compared to those treated with untreated β-chitosan or saline solution controls. Furthermore, analysis of inflammatory cytokine expression levels demonstrated reduced inflammation in the treatment group relative to controls based on RT-PCR. These findings suggest that β-Ch-ZnO-NPs have potential anti-inflammatory and wound-healing-promoting effects in zebrafish, highlighting their therapeutic potential in biomedical applications.

Scanning electron microscopy analysis

**Figure 1:**
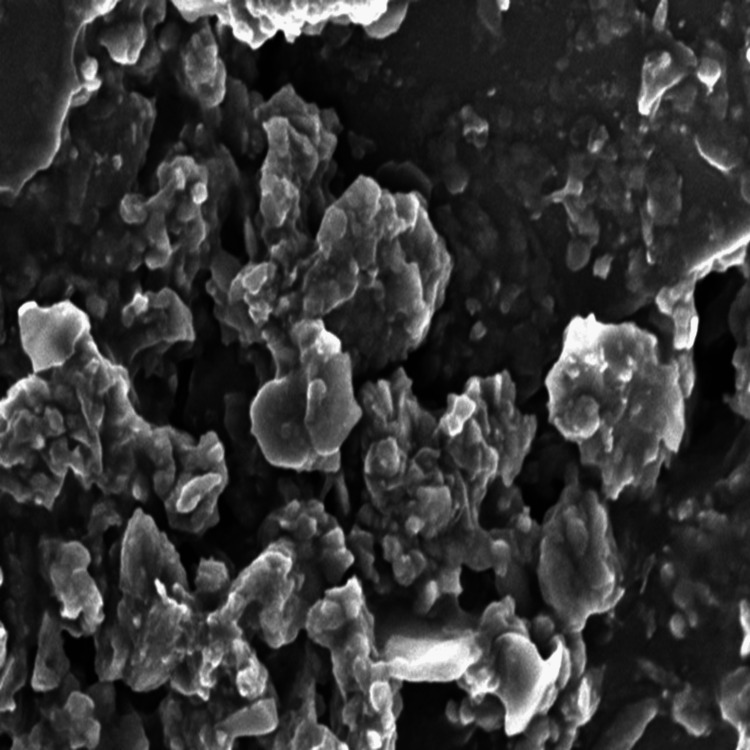
Scanning electron microscopy (SEM) image of β-chitosan-derived zinc oxide nanoparticles. The SEM image depicted the morphology and the size distribution of the synthesized chitosan-derived zinc oxide nanoparticle.

The scanning electron microscopy (SEM) images of β-Ch-ZnO-NPs revealed (Figure 1) the following key characteristics. Nanoparticles were predominately spherical in shape with significant irregularities indicating the successful encapsulation of chitosan particles. The size distribution appeared to be relatively uniform across the sample. The nanoparticles were well dispersed, with minimal science of clumping, indicating a stable colloidal formulation. It was further analyzed by different spectroscopic techniques.

UV-vis spectroscopy analysis

**Figure 2:**
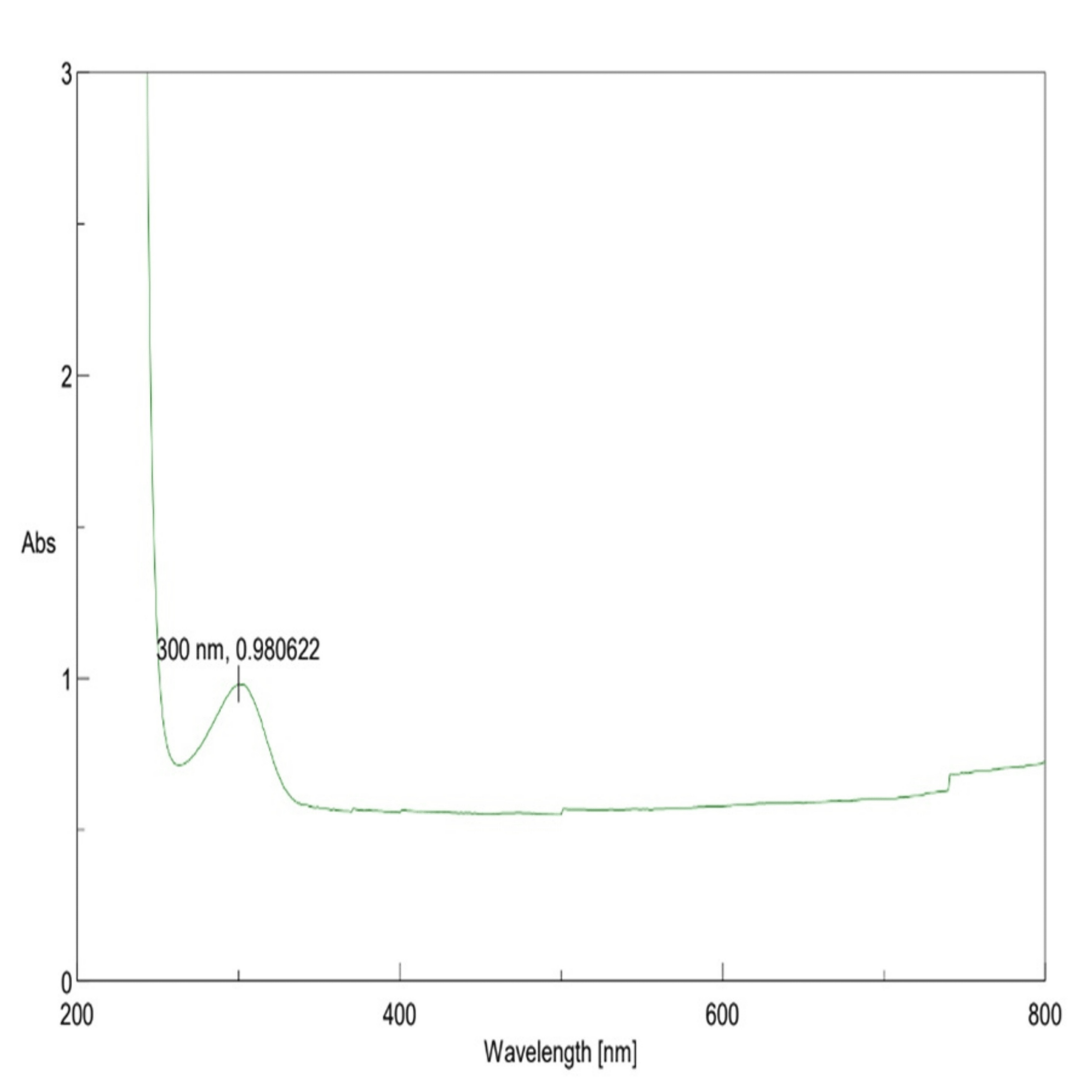
Ultraviolet-visible (UV-vis) absorption spectra of β-chitosan-derived zinc oxide nanoparticles.

Biogenic β-Ch-ZnO-NPs were characterized using UV-visible spectroscopy, revealing a distinct absorption peak at 377 nm. This absorption peak closely matches the bulk exciton absorption of β-Ch-ZnO-NPs, indicating the formation of spherical nanoparticles with an average size range of 40-60 nm. The rapid increase in absorbance upon excitation from the nanoparticle's ground state to its excited state further verifies its optical properties. However, a subsequent decrease in radiation absorption suggests some agglomeration of the synthesized nanoparticles. The bandgap energy of β-Ch-ZnO-NPs was determined to be 3.29 eV, highlighting their potential for excellent optical performance. These findings underscore the successful synthesis of biogenic β-Ch-ZnO-NPs (Figure 2).

FTIR analysis

**Figure 3:**
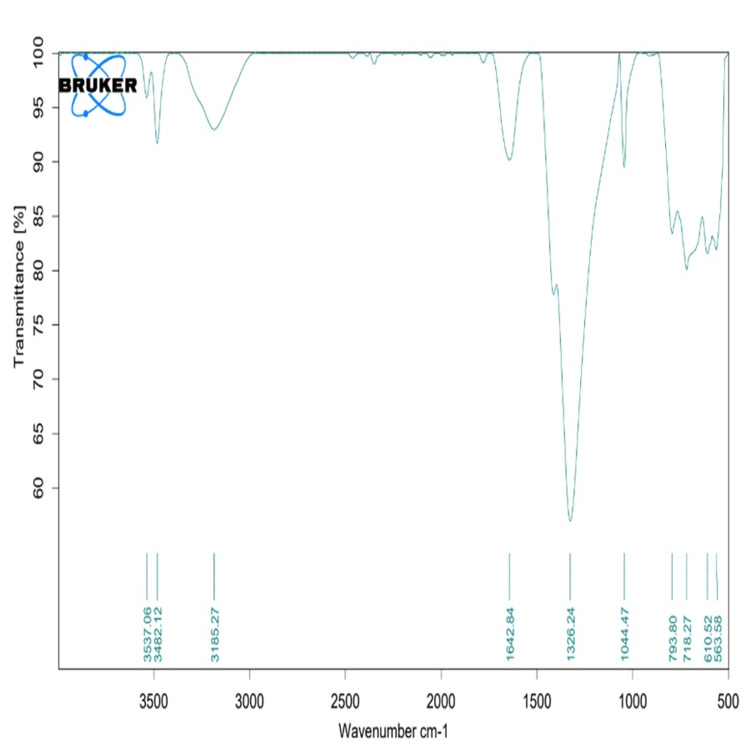
Fourier transform infrared spectroscopy (FTIR) spectra of β-chitosan-derived zinc oxide nanoparticles.

Fourier transform infrared spectroscopy (FTIR) analysis showed the presence of different functional groups on the surface of the synthesized nanoparticle. The peaks such as 3537, 3482, and 3185 showed the presence of stretching vibrations of hydroxyl (OH) and amine (NH-groups). The absorption bands such as 1326 and 1044 showed the presence of CH3 and CH2 groups with bending vibrations from β-chitosan. Further, absorbance at the 1044 band showed Zn-O stretching vibrations, indicating the formation of zinc oxide (ZnO) with β-chitosan. The absorbance band at 793 showed the association of Zn-OH bending vibrations supported by the presence of ZnO nanoparticles (Figure 3). Hence the FTIR analysis confirmed the successful presence of β-Ch-ZnO-NPs (Figure 2).

XRD analysis

**Figure 4:**
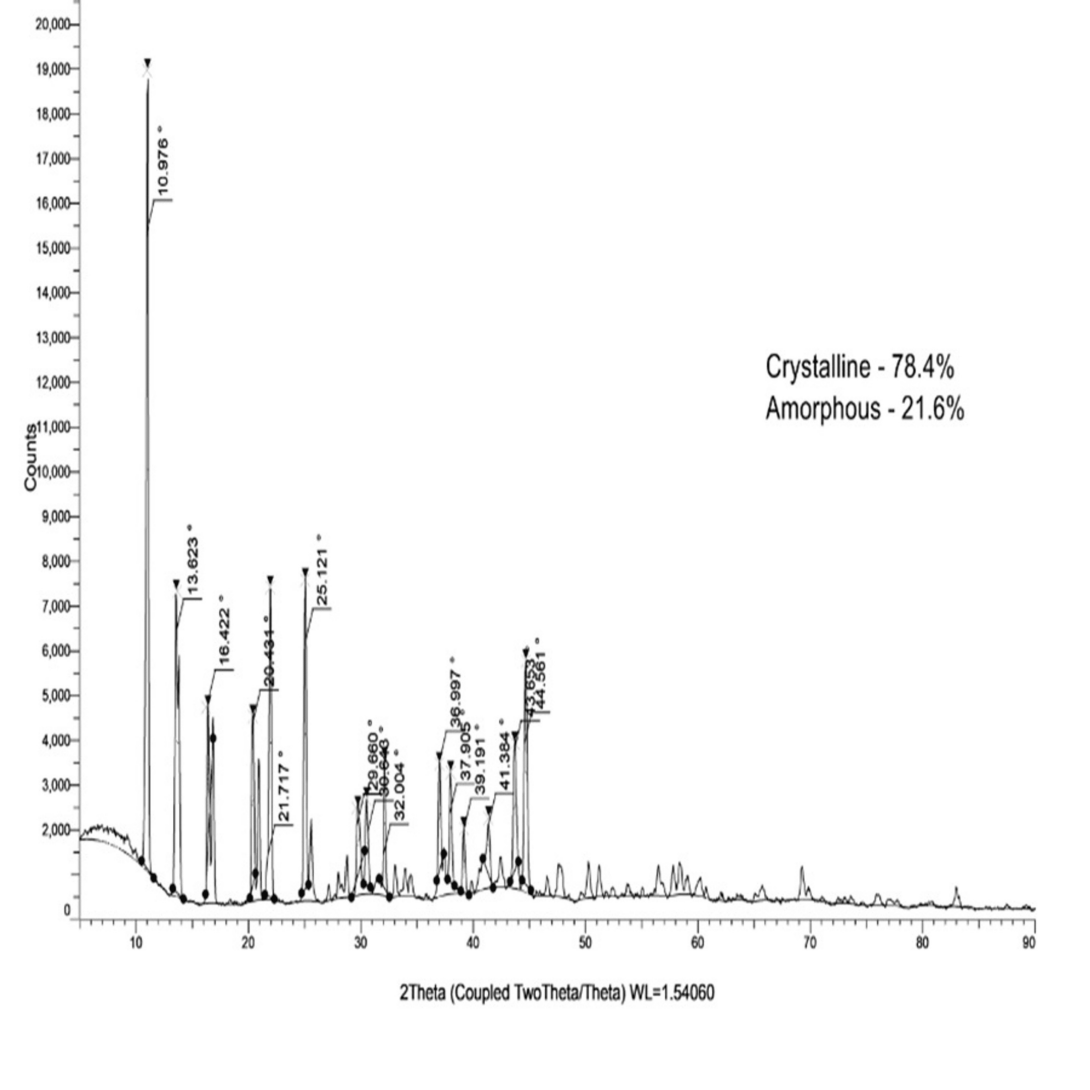
X-ray diffraction (XRD) pattern of as-prepared and annealed (800°C) β-chitosan-derived zinc nanoparticles.

Diffraction patterns from the as-prepared and annealed β-Ch-ZnO-NPs samples were analyzed using Bragg’s law, nλ=2dsin⁡θnλ = 2d \sin θnλ=2dsinθ, where nnn is an integer, λλλ is the wavelength of X-ray radiation, ddd is the interplanar spacing, and θθθ is the diffraction angle. The X-ray diffraction (XRD) analysis of the as-prepared β-Ch-ZnO-NPs revealed distinct diffraction peaks characteristic of the crystalline zinc oxide phase, with specific lattice parameters. No diffraction peaks corresponding to unreacted zinc, zinc oxides, or other phases were observed, indicating the formation of pure zinc oxide nanoparticles. The crystallinity (78.4%) and amorphous (21.6%) showed the presence of predominant crystalline structures of β-Ch-ZnO-NPs than the amorphous structure (Figure 4).

Wound-healing observation

**Figure 5:**
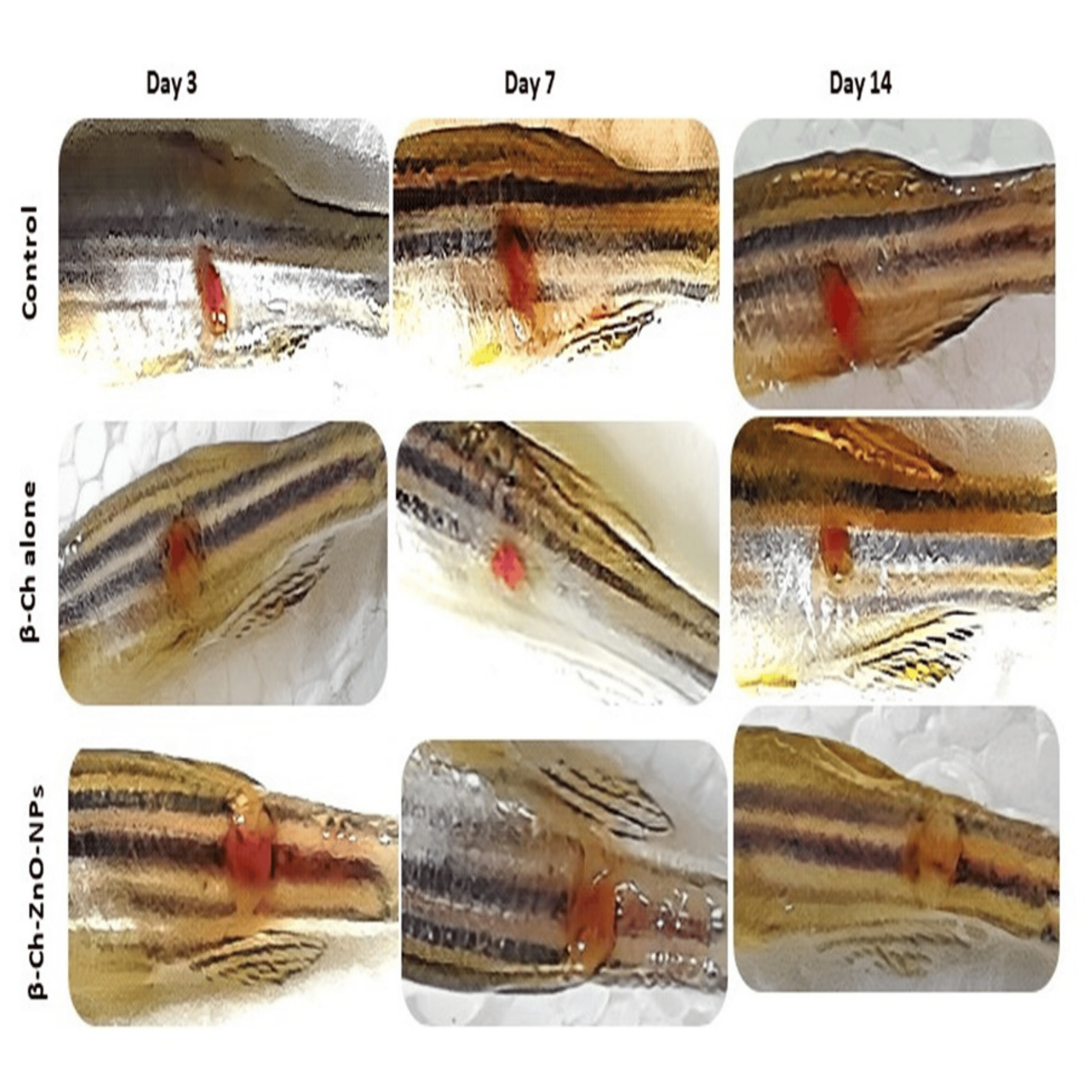
β-chitosan-derived zinc oxide nanoparticles enhance wound healing in zebrafish. Zebrafish were superficially wounded and treated with respective compounds and observed for 15 days (control - phosphate buffer saline, β-chitosan alone (30 µg/ ml), and β-Ch-ZnO-NPs (30 µg/ ml). β-Ch: β-chitosan; β-Ch-ZnO-NPs: β-chitosan-derived zinc oxide nanoparticles. Image credit: Meenakshi Sundaram.

In comparing the treated groups, zebrafish treated with β-Ch-ZnO-NPs exhibited markedly smaller wound sizes compared to the control group. Zebrafish in the nanoparticle-treated group demonstrated accelerated wound healing, characterized by visibility-reduced wound areas over the observation period. These findings suggest that β-Ch-ZnO-NPs effectively promote wound closure and tissue regeneration in zebrafish models. The images provided clear visual evidence of the therapeutic potential of β-Ch-ZnO-NPs in enhancing wound-healing processes, highlighting their promising application in biomedical research and clinical settings (Figure 5).

**Figure 6:**
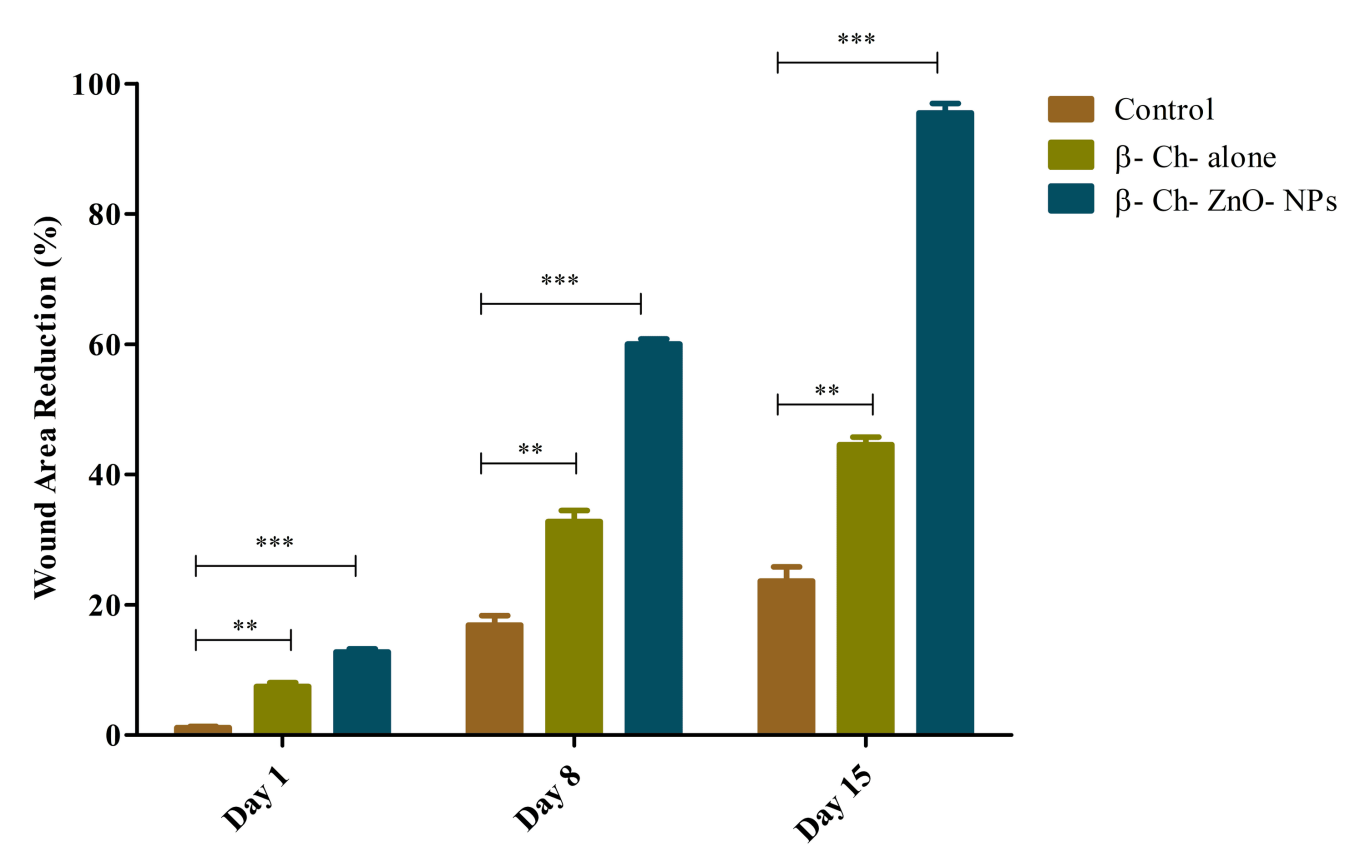
Evaluation of wound-healing activity in zebrafish was carried out in distinct groups compared to the control group. The treatment group exhibited significantly reduced wound size on zebrafish in β-chitosan-derived zinc nanoparticles-treated group in comparison to the control group (Group 1 - β-chitosan-derived zinc nanoparticles; Group 2 - β-chitosan alone). The data were represented as mean ± standard deviation, two-way ANOVA, and Bonferroni post-tests. The results were statistically significant (p < 0.05). P-value was indicated as ** p < 0.01 and *** p < 0.001. Control - phosphate buffer saline, β-chitosan alone (30 µg/ml), and β-Ch-ZnO-NPs (30 µg/ml). β-Ch: β-chitosan; β-Ch-ZnO-NPs: β-chitosan-derived zinc oxide nanoparticles. Image credit: Meenakshi Sundaram

The study tracked the effectiveness of β-Ch-ZnO-NPs, β-chitosan alone, and untreated control over a 15-day period. Initially, control group exhibited <1.5% efficacy on day one. β-Ch-ZnO-NPs treatment showed notable wound area reduction from day one onwards compared to β-Ch- alone. By day eight, the untreated control showed a significant decrease to 35%, whereas β-chitosan-derived zinc nanoparticles maintained 70% efficacy and β-chitosan alone dropped to 60%. By day 15, the untreated control improved slightly to 55%, β-chitosan-derived zinc nanoparticles decreased to 30%, and β-chitosan alone dropped notably to 15% efficacy. These findings indicate that while both treatments initially outperformed the untreated control, the efficacy of β-chitosan-derived zinc nanoparticles diminished over time, while β-chitosan alone showed a steep decline, suggesting varying effectiveness of these treatments in the experimental context (Figure 6). Statistical analysis showed that the differences between the control and β-Ch- alone (p < 0.01) and control and β-Ch-ZnO-NPs (p < 0.001) were significant as indicated by ** and ***, respectively.

Bax expression on wound-healing process

**Figure 7:**
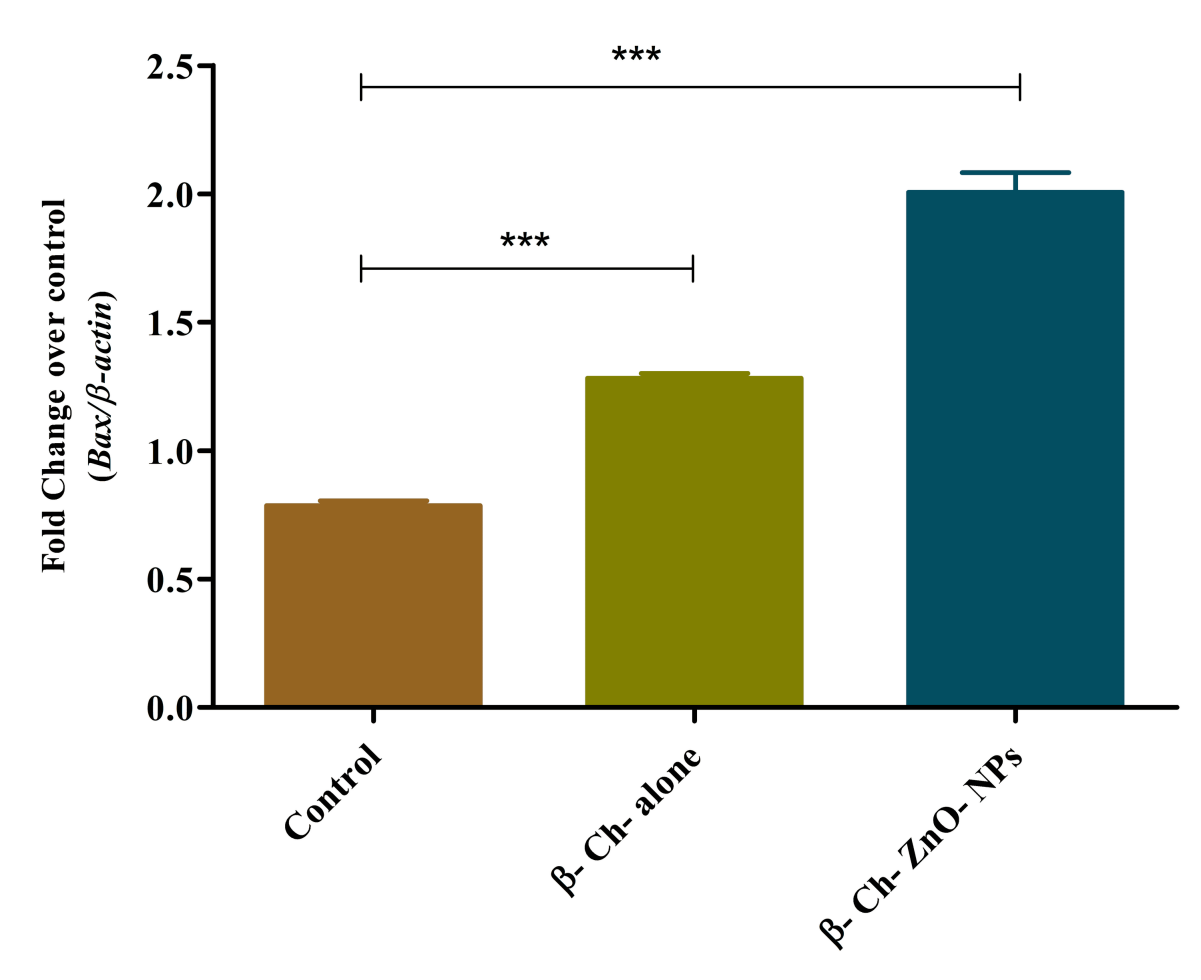
β-chitosan-derived zinc oxide nanoparticles increased Bax expression on wound-healing process in a concentration-dependent manner. The data were represented as mean ± standard deviation, one-way ANOVA, and Dunnett's multiple comparison tests. The results were statistically significant (p < 0.05). P-value was indicated as ** p < 0.01 and *** p < 0.001. Control - phosphate buffer saline, β-chitosan alone (30 µg/ml), and β-Ch-ZnO-NPs (30 µg/ml). β-Ch: β-chitosan; β-Ch-ZnO-NPs: β-chitosan-derived zinc oxide nanoparticles. Image credit: Meenakshi Sundaram

β-Ch-ZnO-NPs influence the expression of Bax, a gene involved in programmed cell death during the wound-healing process. Specifically, it indicates that as the concentration of these nanoparticles increases, so does the expression of Bax. This concentration-dependent effect implies that higher concentrations of β-Ch-ZnO-NPs likely promote or enhance the activation of Bax, which could potentially influence the cellular processes involved in wound healing (Figure 7).

BCl-2 expression on wound-healing process

**Figure 8:**
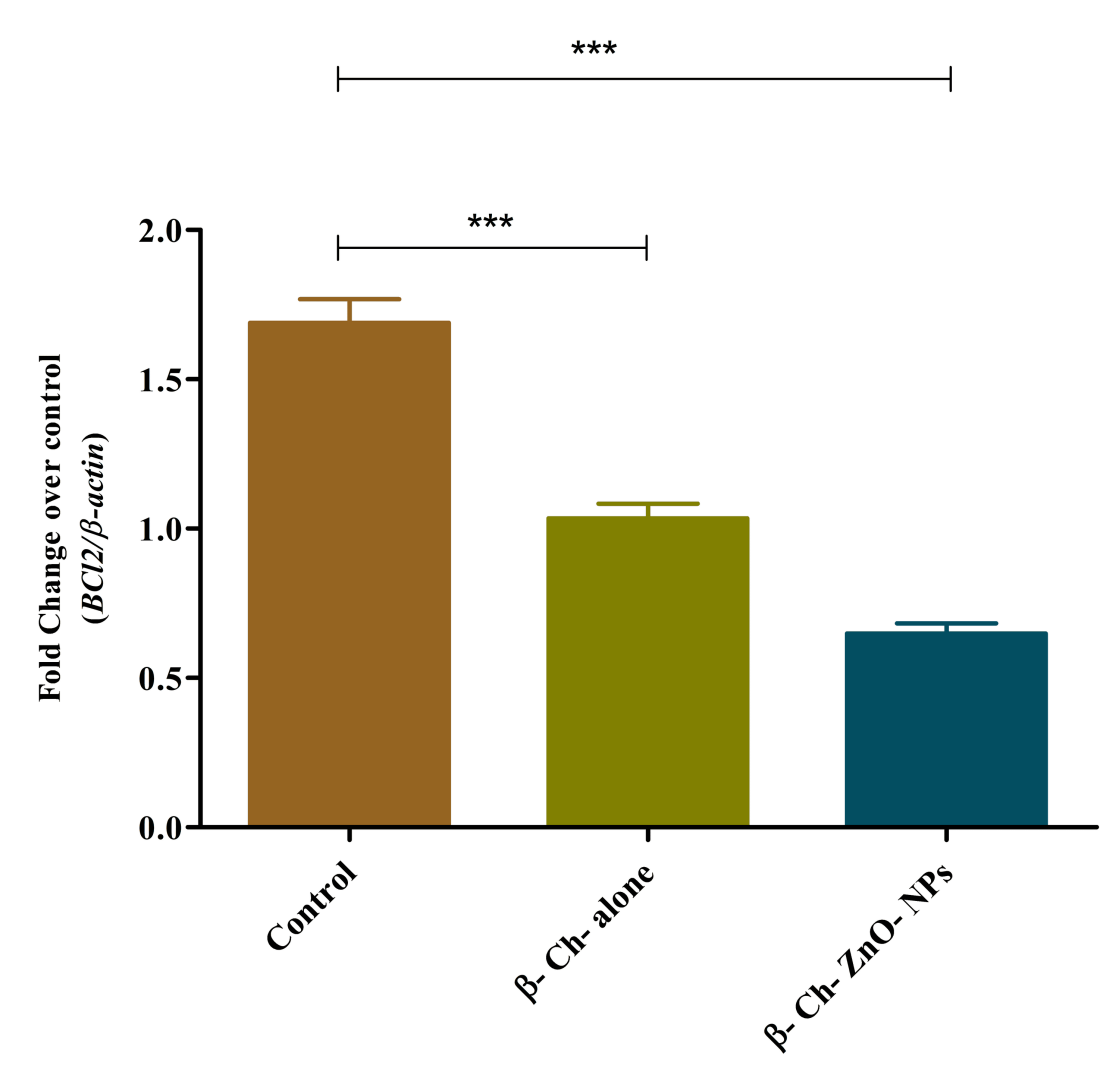
β-chitosan-derived zinc oxide nanoparticles decreased BCl-2 expression on wound-healing process in a concentration-dependent manner. The data were represented as mean ± standard deviation, one-way ANOVA, and Dunnett's multiple comparison tests. The results were statistically significant (p < 0.05). The p-value was indicated as *** p < 0.001. Control - phosphate buffer saline, β-chitosan alone (30 µg/ml), and β-Ch-ZnO-NPs (30 µg/ml). β-Ch: β-chitosan; β-Ch-ZnO-NPs: β-chitosan-derived zinc oxide nanoparticles. Image credit: Meenakshi Sundaram

β-Ch-ZnO-NPs have an impact on the expression ofBCl-2, a gene known for its role in regulating cell survival, in the context of wound healing. Specifically, as the concentration of these nanoparticles increases, the expression of BCl-2 decreases in a concentration-dependent manner. This suggests that a higher concentration of β-Ch-ZnO-NPs may suppress BCl-2 expression, potentially influencing cellular processes that contribute to wound healing (Figure 8).

IL-2 expression on wound-healing process

**Figure 9:**
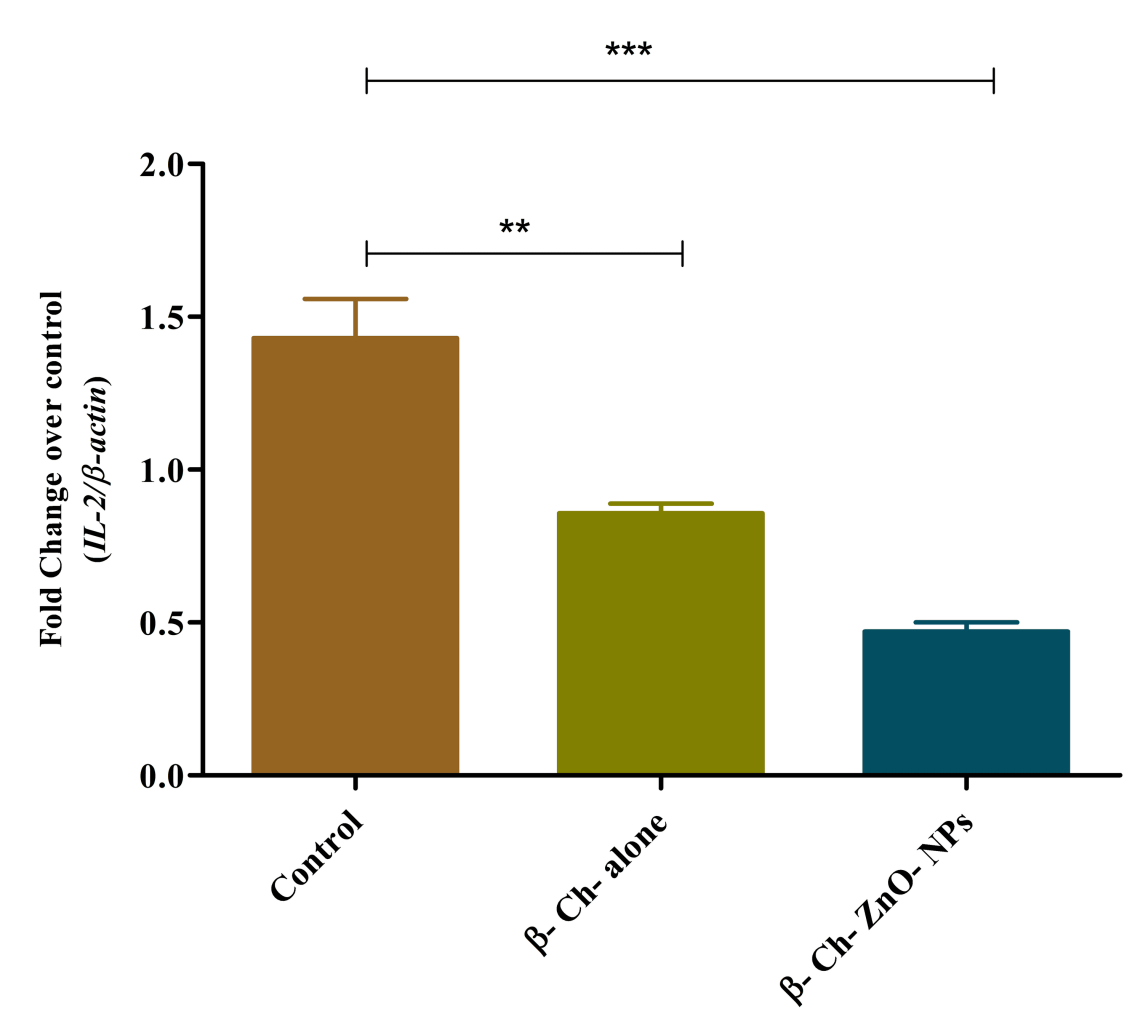
β-chitosan-derived zinc oxide nanoparticles increased lL-2 expression on wound-healing process in a concentration-dependent manner. The data were represented as mean ± standard deviation, one-way ANOVA, and Dunnett's multiple comparison tests The results were statistically significant (p < 0.05). P-value was indicated as ** p < 0.01 and *** p < 0.001. Control - phosphate buffer saline, β-chitosan alone (30 µg/ml), and β-Ch-ZnO-NPs (30 µg/ml). β-Ch: β-chitosan; β-Ch-ZnO-NPs: β-chitosan-derived zinc oxide nanoparticles. Image credit: Meenakshi Sundaram

β-Ch-ZnO-NPs affect the expression of interleukin-2 (IL-2) during the wound-healing process in a concentration-dependent manner. IL-2 is a cytokine that plays a critical role in immune response and cell proliferation. The data indicate that increasing concentrations of these nanoparticles lead to a corresponding increase in IL-2 expression levels. This concentration-dependent effect implies that β-Ch-ZnO-NPs may stimulate the production of IL-2, potentially enhancing immune response and cellular proliferation mechanisms involved in wound healing (Figure 9).

IL-6 expression on wound-healing process

**Figure 10:**
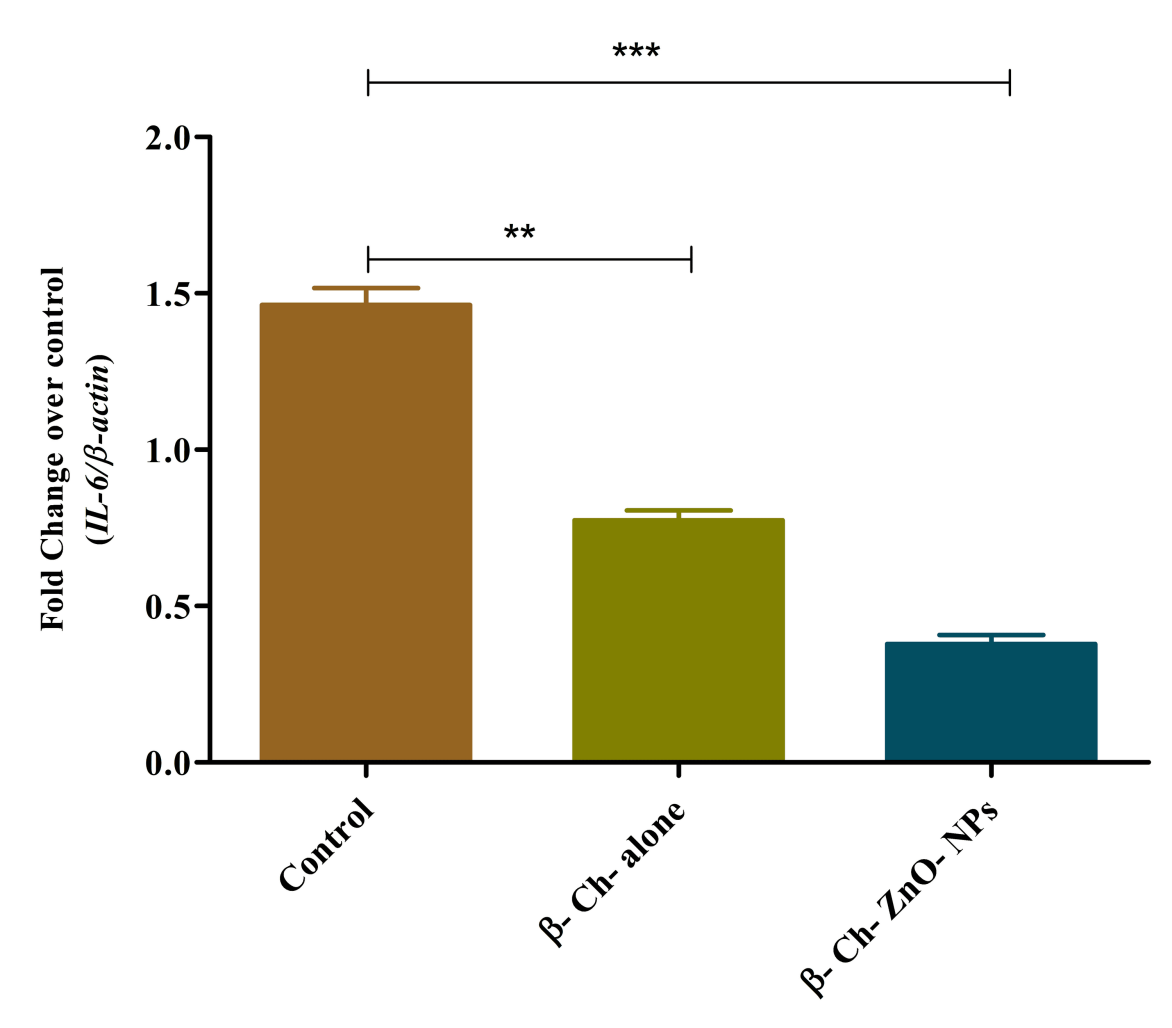
β-chitosan-derived zinc oxide nanoparticles decreased lL-6 expression on wound-healing process in a concentration-dependent manner. The data were represented as mean ± standard deviation, one-way ANOVA, and Dunnett's multiple comparison tests. The results were statistically significant (p < 0.05). P-value was indicated as ** p < 0.01 and *** p < 0.001. Control - phosphate buffer saline, β-chitosan alone (30 µg/ml), and β-Ch-ZnO-NPs (30 µg/ml). β-Ch: β-chitosan; β-Ch-ZnO-NPs: β-chitosan-derived zinc oxide nanoparticles. Image credit: Meenakshi Sundaram

β-Ch-ZnO-NPs have a concentration-dependent effect on IL-6 expression during the wound-healing process. IL-6 is a cytokine involved in inflammation and immune response regulation. According to the data, increasing concentrations of these nanoparticles lead to a decrease in IL-6 expression levels. This indicates that higher concentrations of β-Ch-ZnO-NPs may suppress the production of IL-6, potentially modulating inflammatory responses during wound healing. Understanding this relationship is essential for assessing the nanoparticle's ability to regulate immune signaling pathways and inflammation, which are crucial factors in promoting efficient wound repair. Further investigation into the precise mechanisms by which these nanoparticles influence cytokine expression could provide insights into their therapeutic potential for managing inflammatory conditions and enhancing wound-healing outcomes (Figure 10).

TNF-a expression on wound-healing process

**Figure 11:**
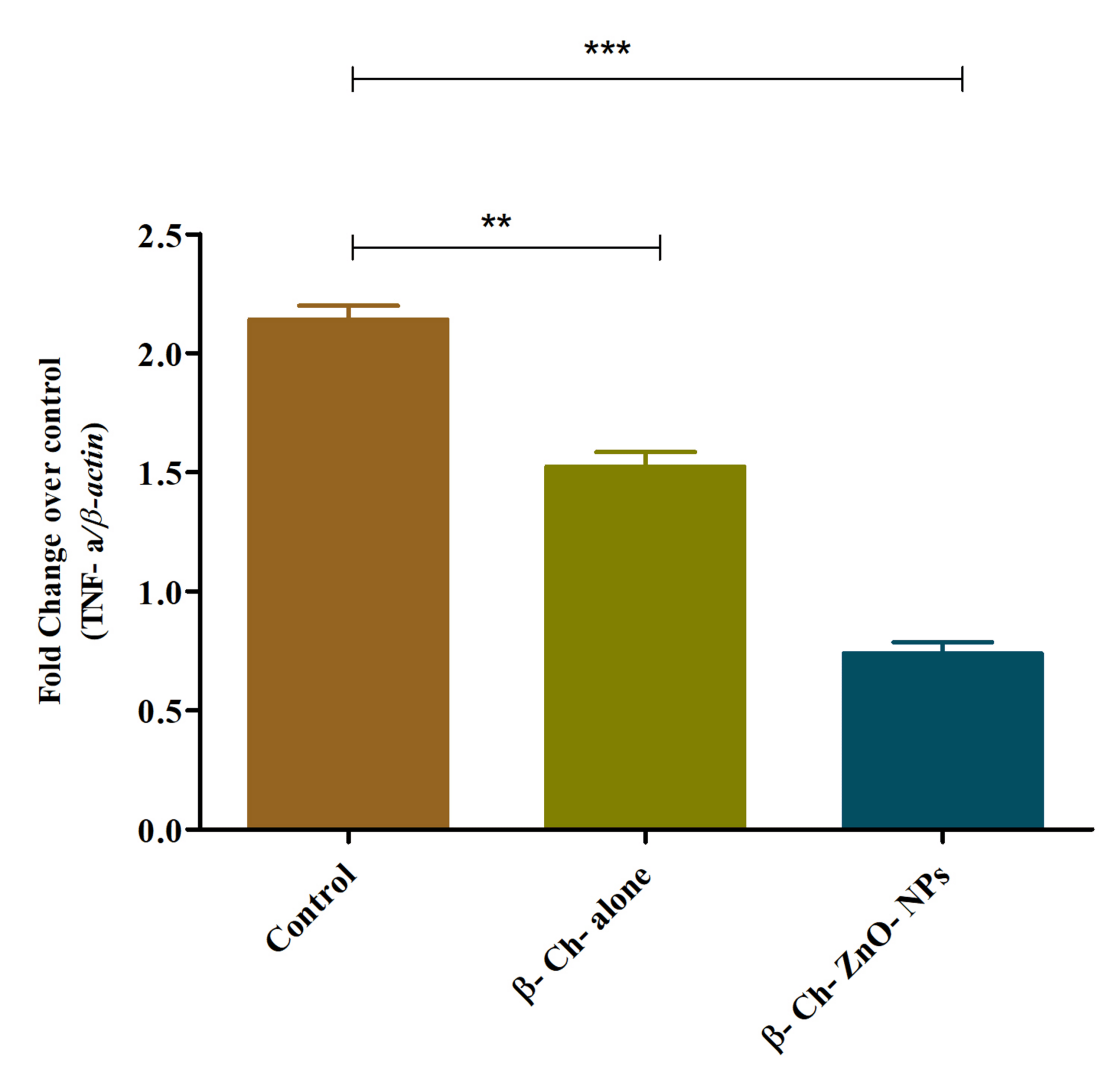
β-chitosan-derived zinc oxide nanoparticles decreased TNF-a expression on wound-healing process in a concentration-dependent manner. The data were represented as mean ± standard deviation, one-way ANOVA, and Dunnett's multiple comparison tests. The results were statistically significant (p < 0.05). P-value was indicated as ** p < 0.01 and *** p < 0.001. Control - phosphate buffer saline, β-chitosan alone (30 µg/ml), and β-Ch-ZnO-NPs (30 µg/ml). β-Ch: β-chitosan; β-Ch-ZnO-NPs: β-chitosan-derived zinc oxide nanoparticles; TNF-a: tumor necrosis factor-alpha. Image credit: Meenakshi Sundaram

The statement indicates that β-Ch-ZnO-NPs exert a concentration-dependent effect on TNF-a expression during the wound-healing process. TNF-a is a pro-inflammatory cytokine involved in immune responses and inflammation regulation. According to the findings, increasing concentrations of these nanoparticles result in a reduction of* *TNF-a expression levels. This suggests that higher concentrations of β-Ch-ZnO-NPs may suppress the production of TNF-a, potentially mitigating inflammatory responses associated with wound healing. Understanding this relationship is crucial for evaluating the nanoparticles' therapeutic potential in modulating immune responses and inflammation, which are pivotal in the healing of wounds (Figure 11).

The findings of this study provide insights into the mechanistic pathways through which β-Ch-ZnO-NPs may exert their anti-inflammatory effects, thereby contributing to the understanding of novel treatment strategies for wound healing. The results indicate that treatment strategies with β-Ch-ZnO-NPs lead to a significant reduction in inflammatory markers likeIL-6 and TNF-a in wound models. This suggests that β-Ch-ZnO-NPs possess potent anti-inflammatory properties, potentially mediated through the modulation of pro-inflammatory cytokines and enzymes involved in the inflammatory cascade. The ability of β-Ch-ZnO-NPs to attenuate the inflammatory response in wound models highlights their potential as a promising therapeutic agent for promoting wound healing and mitigating inflammation-associated complications.

## Discussion

In this study, we investigated the wound-healing efficacy of β-Ch-ZnO-NPs compared to β-chitosan, basal level, and control groups. Our findings demonstrate that β-Ch-ZnO-NPs significantly enhance wound closure and tissue regeneration compared to β-chitosan alone, highlighting their potential as a novel therapeutic agent for wound-healing applications. The results clearly indicate that the β-Ch-ZnO-NPs-treated group exhibited the most substantial reduction in wound area and achieved complete wound closure more rapidly than the β-chitosan-treated group. This enhanced healing efficacy can be attributed to several synergistic factors [23]. Firstly, β-chitosan is known for its biocompatibility and bioactivity, creating a favorable microenvironment for cellular proliferation and migration, which are essential processes in wound repair. The addition of ZnO nanoparticles further enhances these properties due to their antimicrobial and anti-inflammatory effects. ZnO nanoparticles generate reactive oxygen species (ROS) and release zinc ions, which disrupt microbial cell membranes and inhibit their growth, thus reducing the risk of infections that can delay healing processes. Moreover, the anti-inflammatory properties of zinc oxide nanoparticles are crucial in promoting efficient wound healing. Inflammation plays a dual role in wound healing, necessary for initiating the repair process but potentially detrimental if prolonged or excessive. The zinc oxide nanoparticles in our study effectively modulated the inflammatory response, as evidenced by a reduced inflammatory phase, thereby promoting faster tissue regeneration and epithelialization. Histological analysis further supported these observations, revealing advanced tissue regeneration characterized by enhanced epithelialization and reduced inflammatory responses in the β-Ch-ZnO-NPs-treated group. The combination of β-chitosan and zinc oxide nanoparticles thus leverages the complementary strengths of both materials, resulting in a synergistic effect that enhances wound-healing outcomes. The observed antimicrobial activity of zinc oxide nanoparticles not only reduces microbial load at the wound site but also prevents potential infections that could impair healing progress [24].

The zebrafish model used in this study provided several advantages for evaluating the wound-healing efficacy of β-Ch-ZnO-NPs. Their high genetic homology to humans also enhances the translatability of findings to clinical applications, making zebrafish a robust model for initial therapeutic evaluations [25]. Moving forward, future studies should focus on elucidating the underlying molecular mechanisms driving the observed effects of β-Ch-ZnO-NPs on wound healing. Understanding these mechanisms will not only deepen our knowledge but also aid in optimizing nanoparticle formulations for enhanced therapeutic efficacy [26,27]. Long-term safety assessments are also crucial to ensure the clinical viability of β-Ch-ZnO-NPs, particularly regarding potential cytotoxic effects and biocompatibility in mammalian models and eventually in human clinical trials. In conclusion, β-Ch-ZnO-NPs hold significant promise as an effective therapeutic strategy for enhancing wound healing. Their demonstrated ability to accelerate wound closure, promote tissue regeneration, and modulate inflammation underscores their potential utility in clinical settings. By harnessing the synergistic properties of β-Ch-ZnO-NPs, this study provides a foundation for developing advanced nanoparticle-based therapies that could improve outcomes for patients with chronic wounds and other tissue repair applications. Further research and development efforts will be pivotal in realizing the full therapeutic potential of β-Ch-ZnO-NPs in wound healing and border biomedical contexts [28].

While this study demonstrates the promising potential of β-Ch-ZnO-NPs in enhancing wound healing, several limitations warrant consideration. Firstly, the use of zebrafish, while valuable for initial therapeutic evaluations, may not fully capture the complexities of wound healing in mammalian systems, including humans. Differences in skin structure, immune response, and tissue regeneration processes between zebrafish and mammals could affect the translatability of the results. Additionally, the study primarily focuses on short-term outcomes; long-term effects and potential chronic toxicity of zinc oxide nanoparticles were not assessed. The specific molecular mechanism through which β-Ch-ZnO-NPs exert their effects on wound healing remains to be fully elucidated. Moreover, the scalability of the nanoparticle synthesis and the consistency of their quality in practical applications need further investigation. Finally, while the study highlights significant benefits, a comprehensive evaluation of the safety, biocompatibility, and efficacy of these nanoparticles in human clinical trials is essential before widespread clinical adoption. These limitations suggest that further research is necessary to address these gaps and ensure the clinical relevance and safety of β-Ch-ZnO-NPs for wound-healing applications [25-28].

## Conclusions

In conclusion, β-Ch-ZnO-NPs demonstrated superior wound-healing efficacy in zebrafish compared to β-chitosan alone. The nanoparticles accelerated wound closure, promoted tissue regeneration, and effectively modulated inflammation, highlighting their potential as a promising therapeutic agent. Leveraging the synergistic properties of β-chitosan and zinc oxide nanoparticles, this study underscores their application in enhancing wound-healing outcomes. Future research should focus on elucidating molecular mechanisms, optimizing formulations, and conducting rigorous safety assessments to advance β-Ch-ZnO-NPs toward clinical use in treating chronic wounds and other biomedical applications.
